# Effect of remote ischaemic conditioning on infarct size and remodelling in ST-segment elevation myocardial infarction patients: the CONDI-2/ERIC-PPCI CMR substudy

**DOI:** 10.1007/s00395-021-00896-2

**Published:** 2021-10-14

**Authors:** Rohin Francis, Jun Chong, Manish Ramlall, Chiara Bucciarelli-Ducci, Tim Clayton, Matthew Dodd, Thomas Engstrøm, Richard Evans, Vanessa M. Ferreira, Marianna Fontana, John P. Greenwood, Rajesh K. Kharbanda, Won Yong Kim, Tushar Kotecha, Jacob T. Lønborg, Anthony Mathur, Ulla Kristine Møller, James Moon, Alexander Perkins, Roby D. Rakhit, Derek M. Yellon, Hans Erik Bøtker, Heerajnarain Bulluck, Derek J. Hausenloy

**Affiliations:** 1 The Hatter Cardiovascular Institute, University College London, London, WC1E 6HX UK; 2 Cardiovascular and Metabolic Disorders Program, Duke-National University of Singapore Medical School, Singapore, Singapore; 3 Department of Cardiology, National Heart Centre, Singapore, Singapore; 4 Biomedical Research Centre, Bristol Heart Institute, National Institute of Health Research (NIHR), University Hospitals Bristol NHS Foundation Trust and University of Bristol, Upper Maudlin St, Bristol, BS2 8HW UK; 5 London School of Hygiene and Tropical Medicine Clinical Trials Unit, London, UK; 6 Rigshospitalet, Department of Cardiology, University of Copenhagen, Copenhagen, Denmark; 7 Division of Cardiovascular Medicine, Radcliffe Department of Medicine, University of Oxford, Oxford, UK; 8 National Institute of Health Research (NIHR) Oxford Biomedical Research Centre, Oxford, UK; 9 British Heart Foundation Centre of Research Excellence, Oxford, UK; 10 Royal Free Hospital London and Institute of Cardiovascular Science, University College London, London, UK; 11 Leeds Institute of Cardiovascular and Metabolic Medicine, University of Leeds, Leeds, UK; 12 Leeds Teaching Hospitals NHS Trust, Leeds, UK; 13 Department of Cardiology, Aarhus University Hospital, Aarhus, Denmark; 14 Department of Clinical Medicine, Aarhus University, Aarhus, Denmark; 15 Department of MR Research Centre, Aarhus University Hospital, Aarhus, Denmark; 16 Department of Cardiology, Barts Heart Centre, Barts Health NHS Trust, London, UK; 17 William Harvey Research Institute, Queen Mary University London, London, UK; 18 Department of Cardiology, Leeds General Infirmary, Leeds Teaching Hospitals NHS Trust, Leeds, UK; 19 National Heart Research Institute Singapore, National Heart Centre, Singapore, Singapore; 20 Yong Loo Lin School of Medicine, National University Singapore, Singapore, Singapore; 21 Cardiovascular Research Center, College of Medical and Health Sciences, Asia University, Taichung, Taiwan

**Keywords:** Cardioprotection, Cardiovascular magnetic resonance, Myocardial infarct size, Remote ischaemic conditioning

## Abstract

The effect of limb remote ischaemic conditioning (RIC) on myocardial infarct (MI) size and left ventricular ejection fraction (LVEF) was investigated in a pre-planned cardiovascular magnetic resonance (CMR) substudy of the CONDI-2/ERIC-PPCI trial. This single-blind multi-centre trial (7 sites in UK and Denmark) included 169 ST-segment elevation myocardial infarction (STEMI) patients who were already randomised to either control (*n* = 89) or limb RIC (*n* = 80) (4 × 5 min cycles of arm cuff inflations/deflations) prior to primary percutaneous coronary intervention. CMR was performed acutely and at 6 months. The primary endpoint was MI size on the 6 month CMR scan, expressed as median and interquartile range. In 110 patients with 6-month CMR data, limb RIC did not reduce MI size [RIC: 13.0 (5.1–17.1)% of LV mass; control: 11.1 (7.0–17.8)% of LV mass, *P* = 0.39], or LVEF, when compared to control. In 162 patients with acute CMR data, limb RIC had no effect on acute MI size, microvascular obstruction and LVEF when compared to control. In a subgroup of anterior STEMI patients, RIC was associated with lower incidence of microvascular obstruction and higher LVEF on the acute scan when compared with control, but this was not associated with an improvement in LVEF at 6 months. In summary, in this pre-planned CMR substudy of the CONDI-2/ERIC-PPCI trial, there was no evidence that limb RIC reduced MI size or improved LVEF at 6 months by CMR, findings which are consistent with the neutral effects of limb RIC on clinical outcomes reported in the main CONDI-2/ERIC-PPCI trial.

## Introduction

Mortality and heart failure in ST-segment elevation myocardial infarction (STEMI) patients reperfused by primary percutaneous coronary intervention (PPCI) remain significant [38]. As such, new treatments that can be administered as adjuncts to PPCI, are needed to reduce myocardial infarct (MI) size, prevent adverse post-infarct left ventricular (LV) remodelling, and reduce the risk of developing heart failure (HF) [13, 21, 27, 29]. In this regard, remote ischaemic conditioning (RIC), in which brief cycles of non-lethal ischaemia and reperfusion are applied to an organ or tissue (including the arm or leg) away from the heart, has been shown to reduce MI size in small and large animal models of acute myocardial ischaemia/reperfusion injury (IRI) [11, 25, 28, 37]. In the clinical setting, the RIC stimulus can be non-invasively applied to the arm or leg using serial inflations and deflations (3–4 × 5 min cycles) of a pneumatic cuff placed on the upper arm or thigh, to induce brief non-lethal cycles of limb ischaemia and reperfusion [33]. Limb RIC has been demonstrated to increase myocardial salvage and reduce MI size by 20–30% (quantified by cardiac biomarkers, myocardial single-photon emission computerized tomography [SPECT] or cardiovascular magnetic resonance [CMR]), when applied as an adjunct to reperfusion in STEMI patients treated by either thrombolysis [49] or PPCI [1, 12, 46], although not all studies have shown a cardioprotective effect on MI size [15, 31, 44]. Furthermore, a few studies [18, 41, 42] have shown that RIC may improve clinical outcomes (death and rehospitalisation for heart failure) in STEMI patients undergoing PPCI, but they were not adequately powered for these hard clinical outcomes. However, the recently published large multi-centre CONDI-2/ERIC-PPCI trial (comprising 5401 STEMI patients), reported that limb RIC applied as an adjunct to PPCI did not reduce rates of cardiac death or rehospitalisation for HF at 12 months [22].

CMR has emerged as the imaging modality of choice for assessing cardioprotective efficacy of novel therapies for reducing MI size and preventing adverse post-infarct LV remodelling in STEMI, as it is the reference standard imaging modality for quantifying MI size, LV dimensions and function [3, 26, 36]. Furthermore, CMR is able to detect prognostically important coronary microvascular complications of acute myocardial IRI such as microvascular obstruction (MVO) and intramyocardial haemorrhage (IMH) [9].

In a pre-planned CMR substudy of the CONDI-2/ERIC-PPCI trial, we investigated the effect of limb RIC on acute and chronic MI size, MVO, IMH, and LV function and volumes. We hypothesized that limb RIC applied as an adjunct to PPCI would reduce MI size, prevent MVO and IMH, and improve LVEF. This would shed some light on whether the main CONDI-2/ERIC-PPCI trial [22] was neutral because of a lack of benefit of RIC to reduce MI size by CMR or whether there was an impact on MI size, which was not sufficient to translate to an improvement in clinical outcomes.

## Methods

This was a pre-planned CMR substudy of the published CONDI-2/ERIC-PPCI trial [22]. The study received ethical approval from regional and National Health Service research ethics committees and was conducted in accordance with the principles of good clinical practice. In the CONDI-2 component of the study, all participants provided written informed consent before randomisation. In the ERIC-PPCI component of the study, all patients provided initial verbal assent before randomisation, which was followed by written informed consent. In the main CONDI-2/ERIC-PPCI trial, patients were randomised to receive either limb RIC or control [22]. Limb RIC was achieved using an automated pneumatic cuff (CellAegis AutoRIC, Toronto, Ontario, Canada) placed on the upper arm, and comprised of four alternating cycles of 5 min inflations to 200 mmHg and 5 min deflations to 0 mmHg. The control group comprised either a sham device (UK) or standard care (Denmark). RIC was initiated before PPCI either in the ambulance (Denmark) or on arrival at the hospital (UK), and the RIC or sham protocols did not delay onset of PPCI. Patient recruitment took place at seven CMR centres (five in the UK and two in Denmark). Patients in the seven CMR substudy centres were approached for participation in the CMR substudy. The London School of Hygiene & Tropical Medicine Clinical Trials Unit (London, UK) coordinated the trial in collaboration with the Cardiology Trial Unit and Department of Clinical Epidemiology of Aarhus University Hospital (Aarhus, Denmark).

Patient inclusion criteria in the CMR substudy was a diagnosis of STEMI and pre-PPCI TIMI flow ≤ 1. Exclusion criteria were contraindications to CMR scanning (ferromagnetic implants, estimated glomerular filtration rate < 30 ml/min/1.73m^2^) or inability to tolerate a CMR scan (e.g. claustrophobia, inability to lie flat or mechanical complications, hemodynamic instability due to ventricular arrhythmias or cardiogenic shock), previous coronary artery bypass graft surgery, myocardial infarction within the previous 30 days, left bundle branch block on ECG, treatment with therapeutic hypothermia, conditions precluding use of RIC (paresis of upper limb, or presence of an arteriovenous shunt), and life expectancy of less than 1 year due to non-cardiac pathology.

### CMR image acquisition

There was a standardised CMR acquisition protocol in place prior to the start of the study and the CMR endpoints analysis were pre-defined prior to unblinding of any data. Patients underwent CMR scans acutely (aiming for days 3 post-PPCI and up to 7 days), and at 6 months post-PPCI. CMR was performed using the following scanners: Siemens 1.5T (Barts Heart Centre, Bristol Royal Infirmary, Royal Free Hospital in the UK, and Copenhagen Hospital in Denmark), Siemens 3.0T (John Radcliffe Hospital, UK), and Philips 1.5T (Leeds General Infirmary, UK and Aarhus Hospital, Denmark). The CMR protocol included cine images, full LV stack acquisitions of native T1, and basal, mid- and apical LV short-axis T2* maps, full LV stack of late gadolinium enhancement (LGE, 10 min following Gadovist bolus [0.1 mmol/kg]). All short-axis maps and LGE images were aligned with the short-axis cine images.

### Study endpoints

The primary endpoint was MI size on the 6 months CMR scan (expressed as % of LV mass). Secondary endpoints included: acute MI size (expressed as % of LV mass), edema-based myocardial salvage index (edema-based area-at-risk quantified by the extent of myocardial edema on T1-maps minus MI size divided by the edema-based area-at-risk), incidence of MVO (on LGE imaging) and incidence of IMH (on T2* maps); LV volumes (LV end diastolic volume, LVEDV; LV end systolic volume, LVESV); LV mass in grams; and LV systolic function (LV ejection fraction [LVEF]).

### CMR image analysis

The CMR images were uploaded to a secure server to enable transfer to the CMR core lab. CMR parameters were analyzed using dedicated software (CVI42, Circle Cardiovascular Imaging, Calgary, Canada). All images were analyzed by two experienced observers (RF and JC), and reviewed by a third experienced senior observer (HB). Our core lab has previously shown excellent inter-observer and intra-observer reproducibility for infarct size measurement [7]. All observers were blinded to the treatment allocation. LV volumes and function were quantified using disk summation method, with papillary muscles included as part of the LV cavity [4]. The AAR was quantified using native T1 mapping on the acute scan using the 2-SD semi-automated technique and MI size was quantified from the LGE images using the 5-SD semi-automated technique as previously described, expressed as the percentage of overall LV mass [5]. MVO was detected as dark cores within the bright areas of LGE and was included as part of the acute MI size and MVO was also quantified in a binary fashion as present or absent. IMH was identified as the hypointense areas within the infarct-related territory with T2* values < 20 ms on at least one of the short-axis T2* maps [6].

### Statistical analysis

Statistical analysis was performed using commercial statistical software (*Stata Statistical Software: Release 15.1*. College Station, TX: StataCorp LLC) according to the pre-defined statistical analysis plan that was produced prior to any unblinding of the treatment codes. Continuous data were described as mean ± standard deviation (SD) or median (interquartile ranges [IQR]) as appropriate. Categorical data were described as frequencies and percentages. Groups were compared for continuous outcomes using linear regression methods using transformation of outcome data where the distribution was clearly non-normal with mean differences and 95% confidence intervals (CIs) presented. Where a suitable transformation could not be found, non-parametric methods using Mann–Whitney *U* test were used. Risk differences were calculated for binary outcomes together with 95% CIs. A post hoc analysis was undertaken in left anterior descending (LAD) STEMI patients.

## Results

### Baseline characteristics

**Fig. 1:**
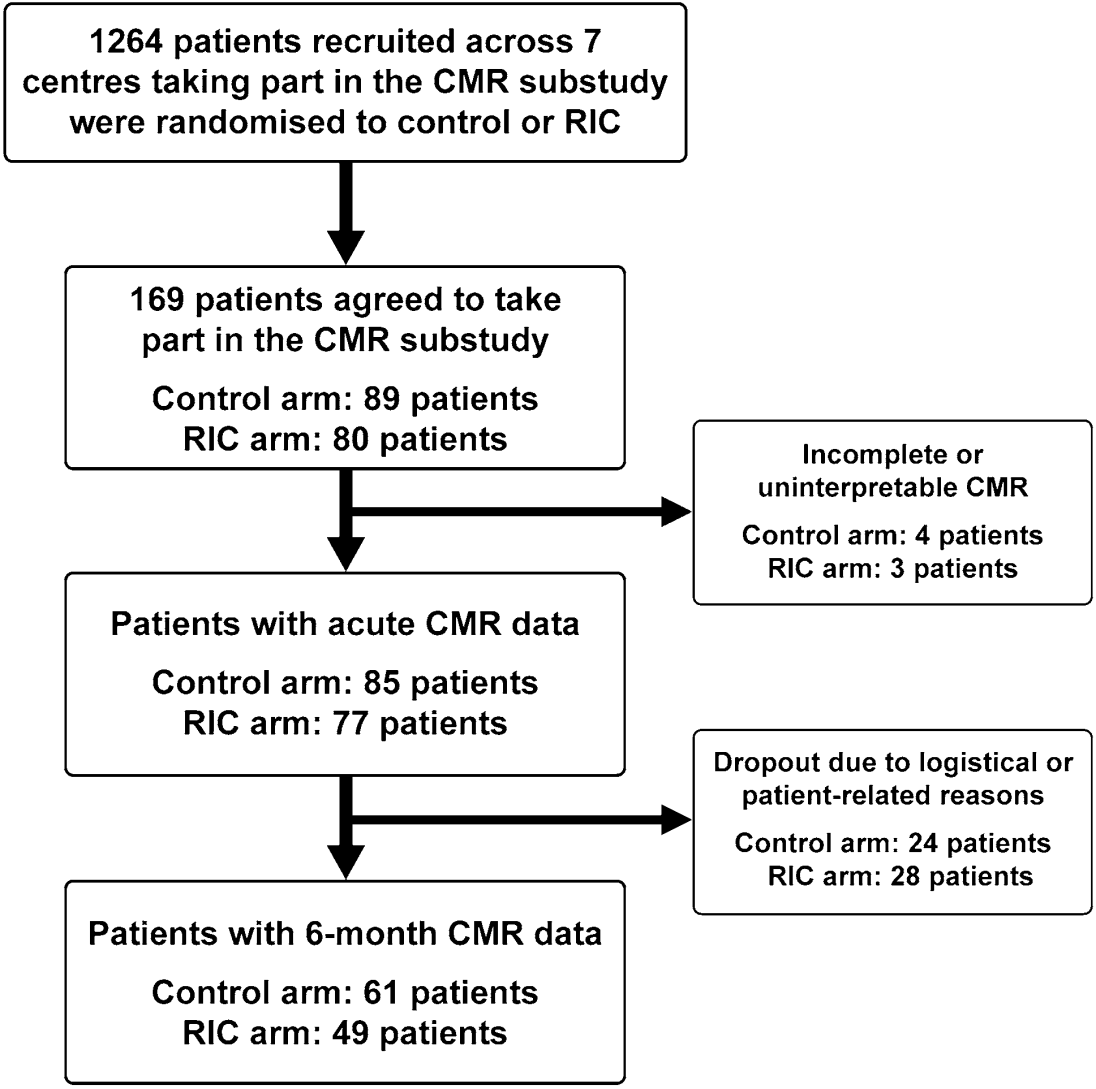
Study flowchart. Study flowchart of the number of patients in each arm undergoing acute and follow-up CMR scans at 6 months

**Fig. 2:**
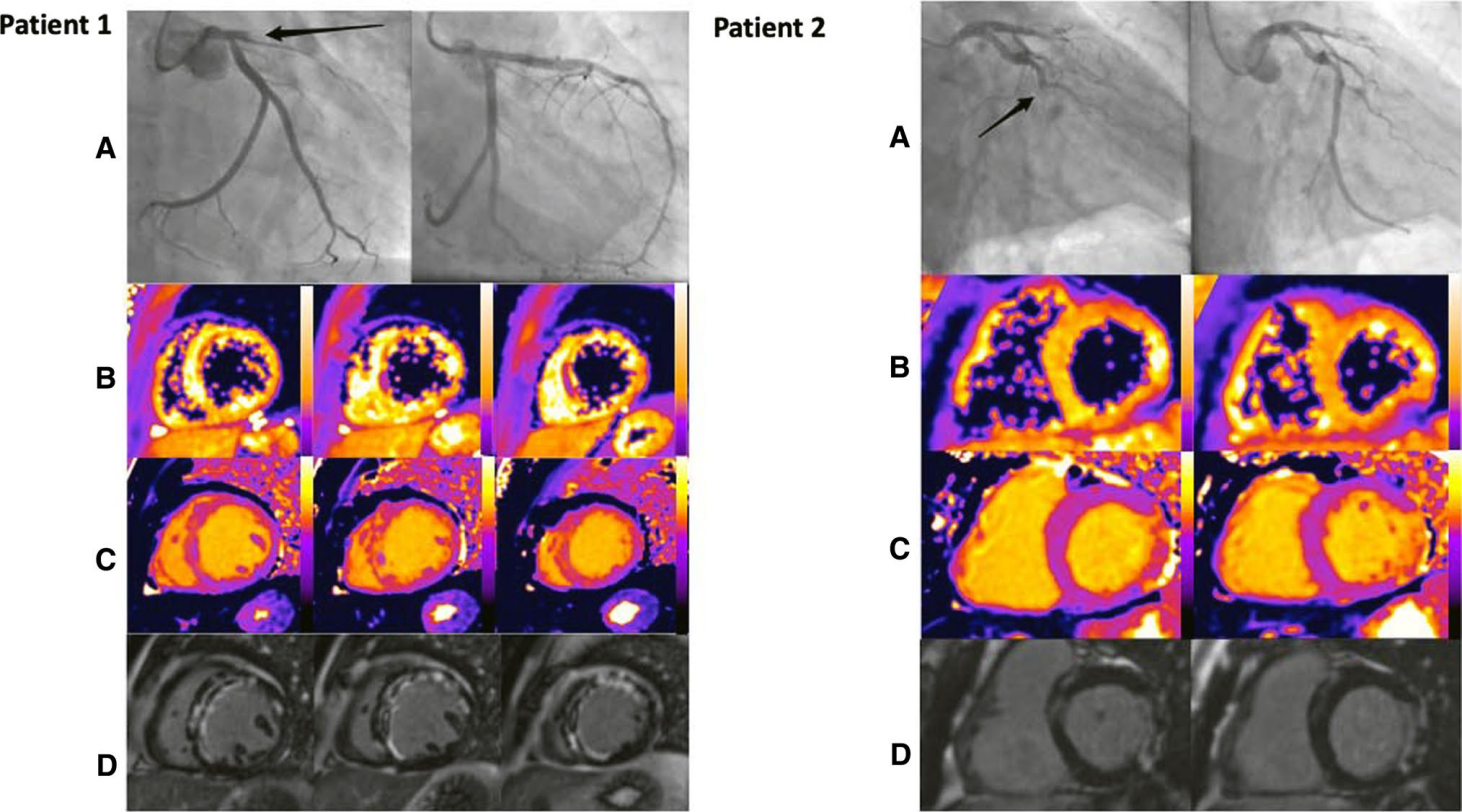
Representative coronary angiography and acute CMR images from 2 CONDI-2/ERIC-PPCI patients. Patient 1. **A** Pre- and post-PCI angiographic images showing a proximally occluded LAD artery, treated successfully by PPCI. **B** Short-axis T2*-maps showing areas of IMH in the anterior wall and septum. **C** Short-axis T1-maps revealing extensive myocardial oedema in the LAD territory with hypointense areas corresponding to areas of MVO and IMH. **D** Short-axis late gadolinium enhancement images showing large area of infarction in LAD territory with MVO. Patient 2. **A** Pre- and post-PCI angiographic images showing an occluded circumflex artery, treated successfully by PPCI. **B** Short-axis T2*-maps shows no evidence of IMH. **C** Short-axis T1-maps revealing myocardial oedema in the lateral wall with hypointense areas corresponding to areas of MVO. **D** Short-axis late gadolinium enhancement images showing a lateral infarct with MVO

1264 patients were recruited at the 7 CMR sites, and of these 169 patients were recruited into the CMR substudy between 7 January 2016 and 26 March 2018, with 162 having analysable acute CMR scans performed at a median of 2 days post-PPCI (interquartile range 1–3 days). Of these, 110 completed the chronic scans performed at 6 months post-PPCI. The flowchart of the patients included in this CMR substudy is shown in Fig. 1, and representative coronary angiography and acute CMR images are provided for two STEMI patients in Fig. 2. The baseline characteristics and PPCI details were reasonably well-balanced between RIC and control (Table 1). Table 2 compares the baseline and procedural characteristics of the patients in the main study with those in the CMR substudy; those in the latter group were more likely to be younger, to be of male sex and more likely to present with RCA territory STEMI.

**Table 1:** Baseline patient characteristics and procedural details

	Control (*N* = 85)	RIC (*N* = 77)
Age (years)	58 ± 12	59 ± 11
Male (%)	80 (94)	68 (88)
BMI (kg/m^2^)	27.2 ± 4.1	27.7 ± 4.1
Smoking status (%)		
Current smoker	35 (41)	32 (42)
Ex-smoker	22 (26)	18 (23)
Never smoked	27 (32)	24 (31)
Unknown	1 (1)	3 (4)
Comorbidities (%)		
Hypertension	34 (41)	29 (38)
Hyperlipidaemia	31 (39)	27 (36)
Ischaemic heart disease	33 (40)	34 (47)
Previous MI	5 (6)	5 (7)
Diabetes Mellitus	9 (11)	8 (11)
Blood pressure (mmHg)		
Systolic	130 ± 28	134 ± 23
Diastolic	78 ± 16	80 ± 13
Killip class (%)		
I	84 (99)	73 (95)
II	0 (0)	2 (3)
III	1 (1)	0 (0)
IV	0 (0)	2 (3)
Infarct-related coronary artery (%)		
Left anterior descending	29 (34)	28 (36)
Circumflex	7 (8)	16 (21)
Right coronary	49 (58)	33 (43)
Chest pain to balloon time (minutes)	176 (120–286)	161 (120–244)
Medical contact to balloon time (minutes)	93 (78–114)	95 (80–124)
Number diseased arteries (%)		
1	47 (55)	39 (51)
2	26 (31)	28 (36)
3	12 (14)	10 (13)
Medications given in relation to PPCI (%)		
Heparin	68 (81)	60 (79)
Aspirin	81 (96)	72 (95)
Clopidogrel	10 (12)	19 (25)
Ticagrelor	76 (90)	63 (83)
Glycoprotein IIb/IIIa inhibitors	19 (23)	17 (22)
Bivalirudin	16 (19)	18 (24)

*RIC* remote ischaemic conditioning; *BMI* body mass index; *MI* myocardial infarction; *PPCI* primary percutaneous coronary intervention

Values are *N* (%), mean ± SD, or median (IQR)

**Table 2:** Baseline patient characteristics and procedural details compared to the main CONDI-2/PPCI study

	CMR substudy (*N* = 162)	Not recruited into CMR substudy (*N* = 4953)
Age/ years	58 ± 11	64 ± 12
Male (%)	148 (91)	3780 (76)
BMI/ kg/m^2^	27.4 ± 4.1	27.5 ± 4.9
Smoking status (%)		
Current smoker	67 (41)	1941 (39)
Ex-smoker	40 (25)	1461 (30)
Never smoked	51 (31)	1372 (28)
Unknown	4 (2)	179 (4)
Comorbidities (%)		
Hypertension	63 (40)	2,057 (42)
Hyperlipidaemia	58 (38)	1,320 (27)
Ischaemic heart disease	67 (43)	1,605 (35)
Previous MI	10 (6)	508 (10)
Diabetes mellitus	17 (11)	550 (11)
Blood pressure/mmHg		
Systolic	132 ± 26	131 ± 24
Diastolic	79 ± 15	76 ± 15
Killip class (%)		
I	157 (97)	4,740 (96)
II	2 (1)	148 (3)
III	1 (1)	24 (< 1)
IV	2 (1)	40 (1)
Infarct-related coronary artery (%)		
Left anterior descending	57 (35)	1,883 (42)
Circumflex	23 (14)	589 (13)
Right coronary	82 (51)	1,958 (44)
Other	0 (0)	18 (< 1)
Chest pain to balloon time/minutes	173 (120–275)	178 (129–278)
Medical contact to balloon time/minutes	94 (79–118)	103 (83–128)
Number of diseased arteries (%)		
0	0 (0)	391 (8)
1	86 (53)	2,615 (54)
2	54 (33)	1,246 (26)
3	22 (14)	572 (12)
Medications given in relation to PPCI (%)		
Heparin	128 (80)	3964 (84)
Aspirin	153 (96)	4498 (95)
Clopidogrel	29 (18)	1229 (26)
Ticagrelor	139 (87)	3148 (67)
Glycoprotein IIb/IIIa inhibitors	36 (23)	830 (18)
Bivalirudin	34 (21)	970 (21)

*CMR* cardiovascular magnetic resonance; *BMI* body mass index; *MI* myocardial infarction; *PPCI* primary percutaneous coronary intervention

Values are *N* (%), mean ± SD, or median (IQR)

### Acute and chronic CMR endpoints

**Fig. 3:**
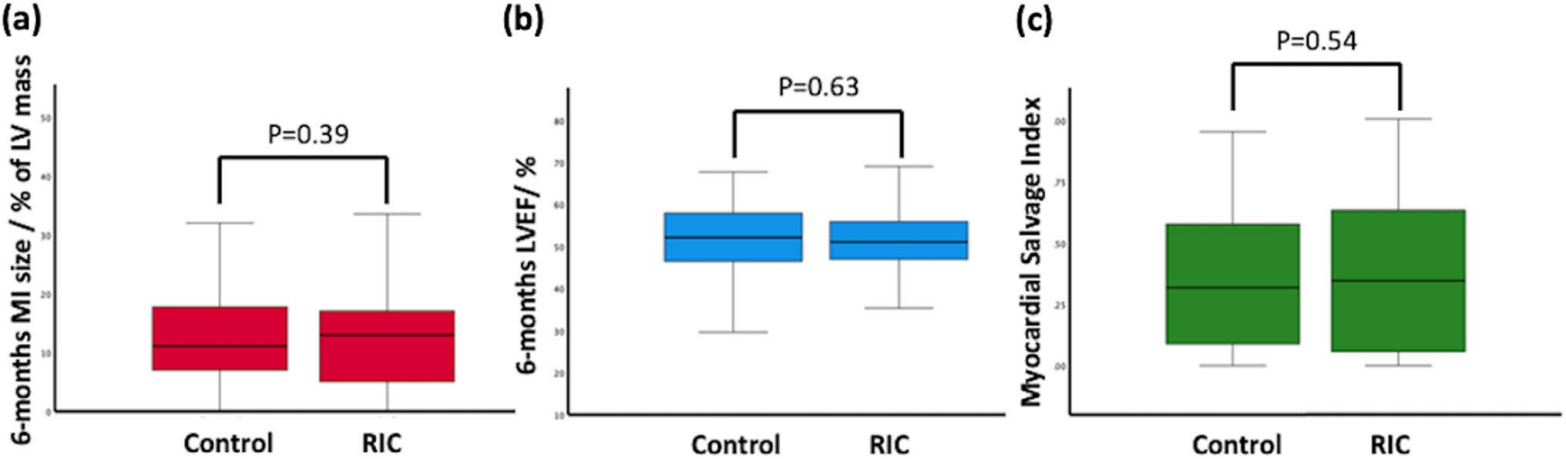
Primary endpoint point and key secondary endpoints. Box plot diagrams of the primary endpoint (6-month MI size) and selected key secondary endpoints (6-month LVEF and edema-based myocardial salvage index on the acute scan)

There was no impact of limb RIC on the primary endpoint of MI size on the 6 months CMR scan [RIC: 13.0 (5.1–17.1)% of LV mass; control: 11.1 (7.0–17.8)% of LV mass, *P* = 0.39, Fig. 3a, Table 3] when compared to control. There was also no difference in LVEF (Table 3), between the RIC group and control. Furthermore, there was also no difference in acute MI size between the RIC group and control (Fig. 3b, Table 3). RIC had no effect on the extent of the edema (Table 3) or LV ejection fraction (Table 3) on the acute CMR scan, when compared to control. There was no difference in the incidence of MVO (RIC 49%; control 61%, *P* = 0.13) and IMH (RIC 36%; control 52%, *P* = 0.067) between RIC and control.

**Table 3:** Effect of limb RIC on CMR outcomes compared to control

	Control	RIC	Difference RIC vs control (95%CI)	*P*-value
Acute CMR scan (*N* = 162)	*N* = 85	*N* = 77		
LVEF/%	49 (43–53)	50 (44–54)	0.8 (−2.0 to 3.7)	0.56
LVESVi/ml m^−2^	44 (37–51)	39 (32–48)	−5.0 (−9.4 to −0.5)	0.20
LVEDVi/ml m^−2^	84 (77–92)	80 (73–90)	−4.0 (−9.0 to 1.0)	0.35
LV mass/g	126 (110–145)	122 (105–137)	−4 (−14 to 5)	0.40
MI size/% of LV mass	18.1 (9.5–27.8)	15.5 (10.0–25.8)	−2.5 (−7.5 to 2.5)	0.32
Edema-based area at risk/% of LV mass	28.0 (19.6–38)	28.1 (19.3–37.7)	0.2 (−4.3 to 4.7)	0.93
Edema-based MSI	0.32 (0.09–0.58)	0.34 (0.06–0.63)	0.04 (−0.10 to 0.18)	0.54
MVO (%)	52 (61)	38 (49)	−12 (−27 to 3)	0.13
IMH (%)	36 (52) *N* = 69	21 (36) *N* = 58	−16 (−33 to 1)	0.067
Chronic CMR scan (*N* = 110)	*N* = 61	*N* = 49		
LVEF/%	52 (47–58)	51 (47–56)	−1.0 (−5.2 to 3.2)	0.63
LVESVi/ml m^−2^	38 (33–50)	39 (33–44)	0.8 (−4.8 to 6.4)	0.74
LVEDVi/ml m^−2^	85 (72–95)	82 (70–93)	−3.3 (−11.2 to 4.6)	0.79
LV mass/g	111 (97–120)	109 (98–123)	−2 (−10 to 6)	0.69
MI size/% LV mass	11.1 (7.0–17.8)	13.0 (5.1–17.1)	1.9 (−2.5 to 6.3)	0.39

*RIC* remote ischaemic conditioning; *CI* confidence interval; *LVEF* left ventricular ejection fraction; *LVESVi* indexed left ventricular end systolic volume; *LVEDVi* indexed left ventricular end diastolic volume; *LV* left ventricular; *MI* myocardial infarct; *MSI* myocardial salvage index; *MVO* microvascular obstruction; *IMH* intramyocardial haemorrhage

Data presented as median (IQR) unless otherwise stated. Treatment effect is absolute risk difference

### LAD and non-LAD STEMI subgroups

In a post hoc analysis of 54 left anterior descending (LAD) STEMI patients, there remained no differences in acute (Table 4) and chronic MI size (Table 4), between the RIC group and control. Of note, LVEF on the acute scan was significantly higher in the RIC group [RIC 49 (42–52)%; control 42 (36–50)%; *P* = 0.041, Table 4], when compared to control. There was also an associated lower incidence of MVO in the RIC group (RIC 52%; control 83%, *P* = 0.012) when compared to control. However, this did not translate to an improvement in LVEF at 6 months (Table 4). There were no differences in the CMR parameters in the non-LAD STEMI subgroup as detailed in Table 4.

**Table 4:** Effect of RIC on CMR outcomes in the subset of LAD and non-LAD STEMI patients

	LAD STEMI				Non-LAD STEMI			
	Control	RIC	Difference RIC vs control (95%CI)	*P*-value	Control	RIC	Difference RIC vs control (95%CI)	*P*-value
Acute CMR scan	*N* = 29	*N* = 25			*N* = 56	*N* = 52		
LVEF/%	42 (36–50)	49 (42–52)	6.7 (0.3 to 13.1)	0.041	51 (46–54)	51 (46–55)	−0.7 (−3.9 to 2.4)	0.64
LVESVi/ml m^−2^	51 (44–59)	43 (36–54)	−7.3 (−16.1 to 1.5)	0.10	41 (35–47)	37 (30–46)	−4.5 (−9.4 to 0.4)	0.069
LVEDVi/ml m^−2^	87 (83–103)	83 (73–92)	−5.5 (−16.8 to 5.9)	0.34	82 (74–89)	78 (71–87)	−2.9 (−8.3 to 2.4)	0.28
LV mass/g	132 (115–159)	126 (108–137)	−6 (−25 to 12)	0.50	122 (109–137)	119 (100–136)	−4 (−15 to 7)	0.50
MI size/% of LV mass	28 (17–41)	22 (10–30)	−6.2 (−19.7 to 7.2)	0.36	15 (7–22)	15 (9–24)	−0.5 (−6.3 to 5.3)	0.86
Edema-based Area at risk/% of LV mass	36 (23–45)	34 (20–54)	2.3 (−15.5 to 20.1)	0.80	26 (18–34)	27 (19–32)	1.8 (−3.3 to 6.9)	0.49
Edema-based MSI	0.11 (0–0.30)	0.20 (0.02–0.59)	0.09 (−0.18 to 0.35)	0.51	0.44 (0.24–0.63)	0.39 (0.10–0.65)	−0.02 (−0.18 to 0.15)	0.83
MVO (%)	24 (83)	13 (52)	−31 (−55 to −7)	0.012	28 (50)	25 (48)	−2 (−21 to 17)	0.84
IMH (%)	18 (69) *N* = 26	10 (44) *N* = 23	−26 (−53 to 1)	0.061	18 (42) *N* = 43	11 (31) *N* = 35	−10 (−32 to 11)	0.34
Chronic CMR scan	*N* = 18	*N* = 15			*N* = 43	*N* = 34		
LVEF/%	49 (42–56)	50 (46–55)	1.4 (−6.6 to 9.5)	0.72	55 (50–58)	52 (48–56)	−2.8 (−7.5 to 1.9)	0.24
LVESVi/ml m^−2^	42 (37–66)	44 (35–59)	1.8 (−16.0 to 19.6)	0.84	37 (32–47)	37 (33–42)	−0.4 (−6.9 to 6.1)	0.89
LVEDVi/ml m^−2^	85 (75–109)	84 (80–108)	−0.8 (−21.5 to 19.8)	0.93	84 (72–94)	77 (70–88)	−7.1 (−18.3 to 3.0)	0.15
LV mass/g	111 (103–114)	108 (99–122)	−3 (−17 to 11)	0.65	111 (97–121)	110 (95–125)	−2 (−14 to 10)	0.74
MI size/% LV mass	19 (12–29)	10 (4–27)	−11.1(−25.7 to 3.6)	0.13	9 (3–15)	13 (6–16)	3.7 (−0.7 to 8.2)	0.10

*RIC* remote ischaemic conditioning; *CI* confidence interval; *LVEF* left ventricular ejection fraction; *LVESVi* indexed left ventricular end systolic volume; *LVEDVi* indexed left ventricular end diastolic volume; *LV* left ventricular; *MI* myocardial infarct; *MSI* myocardial salvage index; *MVO* microvascular obstruction; *IMH* intramyocardial haemorrhage

Data presented as median (IQR) unless otherwise stated. Treatment effect is absolute risk difference

## Discussion

In this pre-planned CMR substudy of the CONDI-2/ERIC-PPCI trial, limb RIC applied as an adjunct to PPCI had no observed beneficial effects on the primary endpoint of chronic MI size at 6 months post-PPCI, or the secondary endpoints of acute MI size, myocardial salvage index or LV ejection fraction when compared to control. Although RIC was associated with less MVO and better acute LVEF in the LAD STEMI subgroup, this did not translate to an improvement in LVEF as 6 months. Overall, these findings are consistent with the neutral effects of limb RIC on clinical outcomes reported in the main CONDI-2/ERIC-PPCI trial [22].

Our study findings do not support the results of previously published RIC studies in STEMI patients [1, 12, 18, 41, 42, 46]. Furthermore, in a subset of 2662 patients in the main CONDI-2/ERIC-PPCI trial, limb RIC did not reduce acute MI size quantified by the 48-h cardiac troponin area-under-the-curve, when compared to control [22] and this is consistent with our CMR substudy findings. However, it is worth noting that serial troponin data were incomplete in the majority of those 2662 patients in the main CONDI-2/ERIC-PPCI trial and there was no surrogate endpoint to account for the area-at-risk [30].

The potential benefit in acute LVEF and MVO seen in the LAD subgroup may have been due to the small sample size and type 1 error. However, there are some supporting evidence to suggest that those patients presenting with a large area-at-risk such as LAD-territory STEMI and with pre-PCI TIMI flow 0 or 1 are more likely to benefit from cardioprotective strategies that are initiated prior to reperfusion [8, 24, 30]. The translation of promising cardioprotective interventions from bench to bedside has proven challenging so far as laboratory experiments are conducted young animals with no comorbidities and focus on mechanistic insights (reductionist model) [40], whereas clinical trials are conducted in a heterogeneous cohort of patients with multiple comorbidities, taking concomitant medications and who may have varying degrees of pre-infarct angina, ischaemic time and anterograde and retrograde flow to the infarct-related territory at presentation. Real-world pragmatic studies have adopted an all-comer approach to facilitate recruitment in a timely fashion and to also make the promising cardioprotective therapy more widely applicable rather than adopting a more selective inclusion criteria of patients most likely to benefit. This approach may potentially dilute the effect size of the intervention [24] and may, in part, account for the neutral results of the CONDI-2/ERIC-PPCI trial [22].

Differences in the study design, patient cohorts recruited, the limb RIC protocol itself, and co-medications administered at the time of PPCI, may, in part, explain the discordant results of our CMR substudy with the published positive studies. Our study was a multi-centre trial across seven sites in UK and Denmark, whereas most of the previously published positive studies were single-centre studies, and may have been subjected to bias. We recruited all-comer STEMI patients with occluded coronary arteries, which may have diluted the cardioprotective effect of limb RIC, with prior studies reporting benefit in patients with larger LAD infarcts [1, 12]. However, two studies which recruited only anterior STEMI patients were also neutral [15, 44]. Furthermore, in the main CONDI-2/ERIC-PPCI trial, there was no difference in clinical outcomes with RIC in the LAD vs non-LAD subgroups. In our substudy, the limb RIC protocol comprised 4 × 5 min arm cuff inflations/deflation mainly delivered on arrival at the hospital, where in some cases, it overlapped with reperfusion, whereas in other studies limb RIC was applied in the ambulance and was completed prior to reperfusion, and may have, therefore, been more effective [1]. However, in the main CONDI-2/ERIC-PPCI trial, there was no difference in clinical outcomes whether limb RIC was delivered in the ambulance or at the hospital. Prior positive studies applied RIC to the leg [12, 18], which may have made RIC more effective given the greater tissue mass [35]. However, two studies applied RIC to the leg and were neutral [15, 44]. The majority of patients in our study were administered the oral P2Y12 inhibitor, ticagrelor prior to PPCI, whereas in the prior positive studies, clopidogrel was predominantly used [1, 12, 46]. Animal studies have reported ticagrelor to have cardioprotective effects [34, 45, 47, 48], and this may have attenuated the benefits of limb RIC in our CMR substudy. However, in the main CONDI-2/ERIC-PPCI trial, there was no difference in clinical outcomes with RIC, when stratified by whether ticagrelor was given or not. Finally, our patient cohort was a low-risk STEMI population comprising > 95% with patients in Killip class I, and our patients received timely and optimal treatment by PPCI, and this may have diminished the cardioprotective effects of RIC [20, 23, 30].

Some studies have shown additive cardioprotective benefits in terms of MI size reduction, increased myocardial salvage, and improve clinical outcomes when limb RIC was combined with other interventions such as morphine [39] or ischaemic postconditioning [14] suggesting that a multi-target approach using combination therapy may be more effective than administering a single cardioprotective intervention. The Remote Ischemic Conditioning With Local Ischemic Postconditioning in High-Risk ST-elevation Myocardial Infarction trial (RIP-HIGH, clinicaltrials.gov identifier: NCT04844931) will further explore this by combining both remote ischemic preconditioning with local ischemic post-conditioning in a high-risk (Killip class ≥ II) STEMI patient. The planned i-RIC trial will investigate the cardioprotective efficacy of a telehealth intervention to monitor compliance in real-time of similar intensive limb RIC protocol following STEMI [50]. Unfortunately, a recent study reported that applying daily episodes of limb RIC (4 × 5-min cycles on the arm) for 1 month, initiated on day 3 post-PPCI, did not reduce MI size or prevent adverse post-infarct LV remodelling at 4 months post-STEMI [43], although in the latter study commencing chronic limb RIC 3 days post-PPCI may have been too late to target key proponents of acute myocardial IRI, to prevent adverse post-infarct LV remodelling.

The main limitations of our study are, first, those in the CMR substudy were younger, more likely to be male and were more likely to present with RCA territory STEMI than those in the main trial. Second, the number of patients who completed the CMR scans was smaller than the planned sample size of 250 patients and, therefore, our study may be underpowered. An important number of patients did not undergo their chronic CMR, but we did not collect information on the specific reasons for these dropouts. The predominant reasons were likely due to a combination of patient-related and logistic reasons. These factors highlight the challenges of using CMR as a surrogate endpoint as only those able to tolerate a CMR scan and survive to 6 months would enter the substudy which creates an element of selection bias. The recent introduction of fast scanning CMR protocols for MI size and LVEF to under 15 min [19] may help to make CMR more tolerable to patients, keep costs down and improve accessibility, all of which may reduce dropout rates in future studies. We have presented data on the edema-based area-at-risk and edema-based myocardial salvage index in Tables 3 and 4 as this was a pre-planned secondary analysis in this substudy. However, recent data have shown that the extent of edema can be dynamic within the first few days of a STEMI [10, 16] and certain cardioprotective therapies that reduce MI size can also reduce the extent of the edema [2, 17]. As a result, the recent Journal of the American College of Cardiology scientific panel consensus document has advised against the use for the extent of edema as a surrogate for the area-at-risk in future studies [32].

In summary, in our pre-planned CMR substudy of the CONDI-2/ERIC-PPCI trial, we found that limb RIC did not reduce acute or chronic MI size or affect post-infarct LVEF, findings which are consistent with the neutral effects of limb RIC on clinical outcomes observed in the main CONDI-2/ERIC-PPCI trial [22]. Whether limb RIC may confer benefit in higher risk STEMI patients such as those presenting with cardiac arrest or cardiogenic shock, or in those countries in which ischaemic times are prolonged and PPCI is not widely available, remains to be tested [30].
